# Neoadjuvant photodynamic therapy augments immediate and prolonged oxaliplatin efficacy in metastatic pancreatic cancer organoids

**DOI:** 10.18632/oncotarget.24425

**Published:** 2018-02-06

**Authors:** Mans Broekgaarden, Imran Rizvi, Anne-Laure Bulin, Ljubica Petrovic, Ruth Goldschmidt, Iqbal Massodi, Jonathan P. Celli, Tayyaba Hasan

**Affiliations:** ^1^ Wellman Center for Photomedicine, Department of Dermatology, Harvard Medical School, Massachusetts General Hospital, Boston, MA 02114, USA; ^2^ Department of Physics, University of Massachusetts, Boston, MA 02125, USA

**Keywords:** translational therapies, organoid models, experimental medicine, combination therapy, photochemotherapy

## Abstract

Effective treatment of advanced metastatic disease remains the primary challenge in the management of inoperable pancreatic cancer. Current therapies such as oxaliplatin (OxPt)-based chemotherapy regimens (FOLFIRINOX) provide modest short-term survival improvements, yet with significant toxicity. Photodynamic therapy (PDT), a light-activated cancer therapy, demonstrated clinical promise for pancreatic cancer treatment and enhances conventional chemotherapies with non-overlapping toxicities. This study investigates the capacity of neoadjuvant PDT using a clinically-approved photosensitizer, benzoporphyrin derivative (BPD, verteporfin), to enhance OxPt efficacy in metastatic pancreatic cancer. Treatment effects were evaluated in organotypic three-dimensional (3D) cultures, clinically representative models that bridge the gap between conventional cell cultures and *in vivo* models. The temporally-spaced, multiparametric analyses demonstrated a superior efficacy for combined PDT+OxPt compared to each monotherapy alone, which was recapitulated on different organotypic pancreatic cancer cultures. The therapeutic benefit of neoadjuvant PDT to OxPt chemotherapy materialized in a time-dependent manner, reducing residual viable tissue and tumor viability in a manner not achievable with OxPt or PDT alone. These findings emphasize the need for intelligent combination therapies and relevant models to evaluate the temporal kinetics of interactions between mechanistically-distinct treatments and highlight the promise of PDT as a neoadjuvant treatment for disseminated pancreatic cancer.

## INTRODUCTION

Advanced pancreatic cancer is a lethal malignancy for which the late diagnosis and limited effective treatment options yield 5-year survival rates below 5% [1]. Standard palliative care of advanced inoperable pancreatic cancer involves systemic gemcitabine, which achieves median patient survival to 5.6 months post-diagnosis [2]. Advances in chemotherapeutic approaches have seen modest success in large placebo-controlled clinical trials [2, 3], the most effective being FOLFIRINOX, a stringent chemotherapy cocktail comprising folinic acid, 5-fluorouracil, irinotecan, and oxaliplatin (OxPt) [1]. However, the high toxicity of FOLFIRINOX renders only patients with high performance scores eligible for this regimen [1, 4]. These statistics illustrate the need for more effective and less toxic treatment strategies for pancreatic cancer. Intelligently designed combination therapies may be crucial to overcome treatment resistance and improve survival, in part by reducing tumor burdens to make more patients eligible for surgery [5].

Recent studies have demonstrated the potential of photodynamic therapy (PDT) for the treatment of pancreatic cancer. PDT for internal solid tumors comprises the systemic administration of a photosensitizing agent that diffuses into the tumor tissue. Subsequent irradiation of the tumor tissue with non-toxic laser light excites the photosensitizer to generate highly cytotoxic reactive molecular species at the tumor site. Tumor tissues are consequently eradicated through excessive oxidative damage, shutdown of the tumor vasculature, hypoxia, hyponutrition, and an anti-tumor immune response [6, 7]. *In vitro*, PDT was highly effective against chemoresistant pancreatic cancer cells [8], and adjuvant PDT was shown to reduce pancreatic cancer dissemination and enhance the efficacy of irinotecan and cabozantinib as a neoadjuvant therapy in orthotopic xenograft mouse models [9, 10]. A recent phase I/II clinical trial demonstrated that PDT was feasible and safe for the (adjuvant) treatment of inoperable pancreatic cancer patients, achieving a median patient survival of 8 months [11]. Importantly, a remarkable reduction in tumor volume following PDT rendered a single inoperable patient eligible for surgery, as the shrinkage of the tumor volume resulted in the superior mesenteric artery to be no longer enveloped by the malignancy [11]. These results demonstrate that PDT is feasible for clinical application as a (neo)adjuvant therapy for pancreatic cancer patients, and that its unique therapeutic mechanism may synergize with conventional chemotherapies for this disease. An unanswered question of critical importance remains the capacity of PDT to enhance treatment responses for disseminated pancreatic cancer metastases.

For investigations towards new treatments and their expedited clinical translation, 3D culture models of cancer are gaining appreciation and are being increasingly utilized. These models recapitulate the architecture and heterogeneity of tumors with more accuracy than conventional 2D cell cultures [12], yet still retain high-throughput in comparison to *in vivo* models [13]. For pancreatic cancer specifically, various studies have employed 3D culture models for a range of investigations [14], including mechanisms of oncogenic transformation [15], redox metabolism in response to treatment [16], effects of stromal components and extracellular matrix rigidity on treatment response [17], and to recapitulate tumor heterogeneity and perform therapeutic screening [18]. Thus, 3D culture models represent a multifunctional preclinical platform that bridge the gap between *in vitro* and *in vivo* experimentation [12].

In the current study, we leverage established 3D culture methods and a recently developed treatment analysis methodology [19] to model micrometastatic pancreatic cancer and investigate the potential of a combination therapy that comprises PDT and subsequent OxPt chemotherapy. The current study builds on the demonstrated ability of PDT to overcome chemoresistance and synergize with conventional cancer treatments, including platinum-based chemotherapies [20–22]. The primary goals were to establish whether neoadjuvant PDT could augment the efficacy of OxPt chemotherapy and whether PDT can prevent tumor recovery in 3D-cultures for micrometastatic pancreatic cancer. The results of this study demonstrate that the intrinsic chemotherapy resistance of AsPC-1 micrometastatic pancreatic cancer organoids can be overcome with neoadjuvant PDT, and that this combination therapy may hold potential in reducing the viability and extent of residual disease of micrometastatic pancreatic cancer.

## RESULTS

### Growth characteristics of 3D cultures comprising primary and metastatic pancreatic cancer cells

To model micrometastatic pancreatic cancer *in vitro*, we applied established protocols for 3D adherent cultures on solidified Matrigel scaffolds [21, 23] to grow tumor organoids from metastatic AsPC-1 human pancreatic cancer cells. To assess the effects of treatment on 3D cultures and optimize the treatment schedules, it is imperative to first characterize the growth behavior of such cultures.

**Figure 1:**
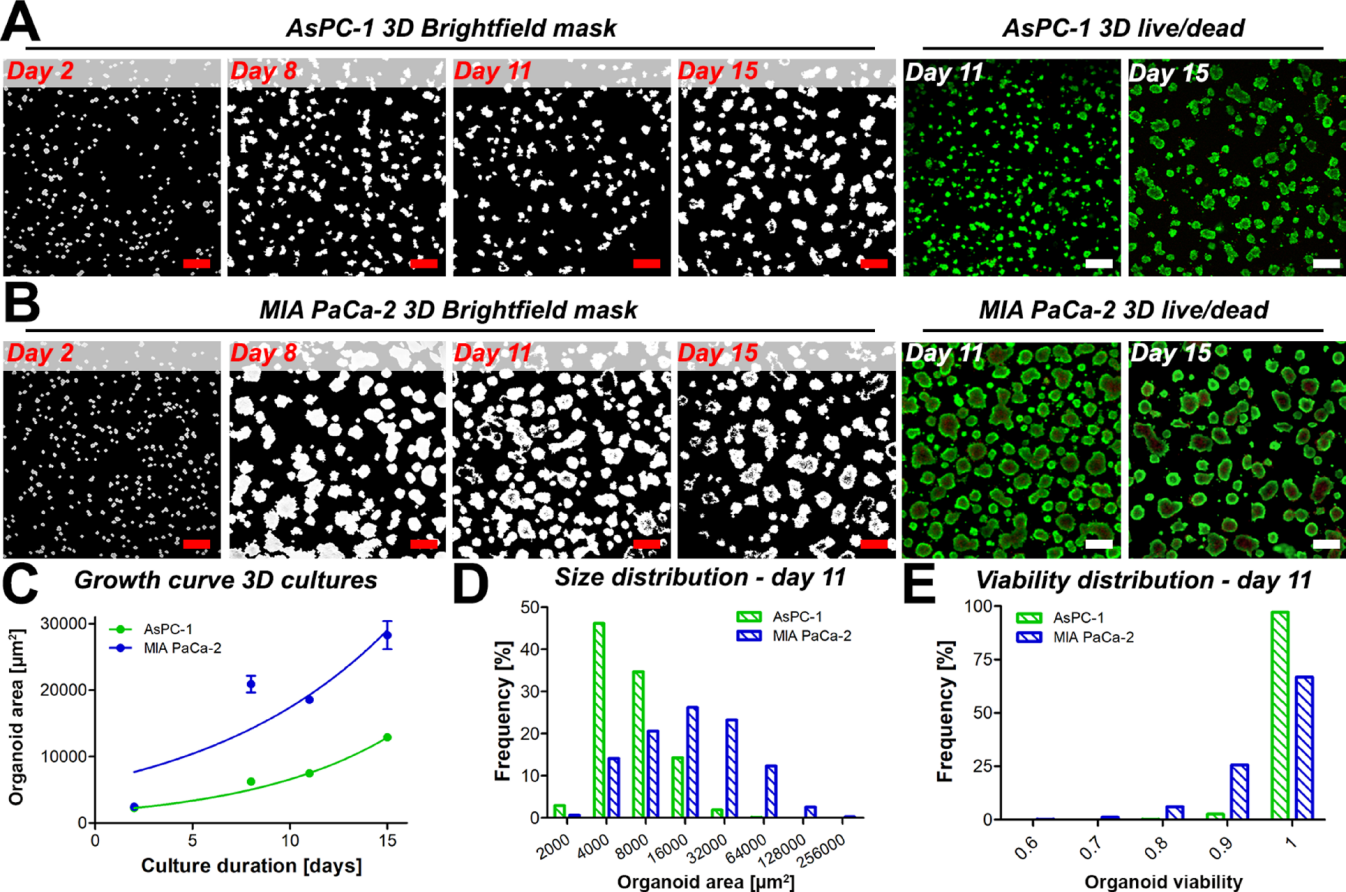
Masked brightfield images illustrate the overall size and spacing of the tumor organoids of AsPC-1 (**A**) and MIA PaCa-2 (**B**) cultures. Cultures were stained for live (calcein AM, green) and dead (PI, red) cells to reveal the presence of necrotic cores in the 3D cultures (live/dead panels) in absence of treatment on days 11 and 15. Scalebar = 400 μm. (**C**) Growth of AsPC-1 (green) and MIA PaCa-2 (blue) organoids (mean ± 95% CI, *N* = 3000–8000). (**D**) Size distributions of individual AsPC-1 (green) and MIA PaCa-2 (red) organoids on day 11, in μm2. (**E**) Distributions of organoid viability of AsPC-1 organoids (green) and MIA PaCa-2 organoids (blue) on culture day 11.

The general growth features of AsPC-1 organoids were investigated and compared to organoid cultures comprising MIA PaCa-2 cells. As specified by the American Type Culture Collection, AsPC-1 cells are derived from the ascites of a 62-year-old female with pancreatic cancer, whereas the epithelial MIA PaCa-2 cells originate from the primary pancreatic tumor of a 65-year old male [24]. In 3D adherent cultures, the cell lines exert clear phenotypic differences (Figure 1A, 1B); it was observed that AsPC-1 cells form substantially smaller organoids compared to organoids composed of MIA PaCa-2 cells. Necrotic cores within individual tumor organoids were typically absent in AsPC-1 organoids (Figure 1A), yet frequently observed in the MIA PaCa-2 cultures (Figure 1B). Organoid growth curves (Figure 1C) illustrate that MIA PaCa-2 tumors expand at a significantly higher rate (*P <* 0.05), corresponding to higher proliferation rates and cell numbers in these cultures (Supplementary Figure 1). Organoid size distributions of both cultures measured 11 days after culture initiation are depicted in Figure 1D and reveal that organoid sizes of AsPC-1 and MIA PaCa-2 cultures are not normally distributed (D’Agostino & Pearson-Omnibus test *p <* 0.0001 for both distributions). Non-parametrical statistical analysis showed that AsPC-1 organoids were significantly smaller compared to MIA PaCa-2 organoids (median size 4.0*10^3^ μm^2^ vs 1.1*10^4^ μm^2^, *p <* 0.001, Mann-Whitney *U*-test). This was recapitulated in separate experiments that compared the growth and size-distributions of MIA PaCa-2, AsPC-1, which additionally included 3D cultures of PANC-1 human primary pancreatic carcinoma cells (Supplementary Figure 2). Lastly, the viability of untreated AsPC-1 organoids was higher and less heterogeneously distributed compared to the viabilities of untreated MIA PaCa-2 organoid cultures, which is attributed to the absence of necrotic cores in the AsPC-1 cultures (Figure 1E). Taken together, the results demonstrate clear variations in tumor growth rates and baseline viabilities, emphasizing the need to normalize organoid size and viabilities to compare treatment effects between cultures of different cell types.

The growth and size characteristics of organoid cultures are relevant with respect to drug diffusion and treatment efficacy in cancer organoids. Previous investigations have demonstrated that platinum-based chemotherapeutics are capable of diffusing throughout entire organoids with higher concentrations at the organoid peripheries [25, 26]. However, studies on ovarian cancer organoids showed that there was a correlation between nodule size and cisplatin efficacy [27]. With respect to PDT, previous studies have demonstrated that BPD has a limited diffusion range that was sufficient to completely photosensitize small organoids, yet photosensitization was restricted to the periphery of larger organoids [28]. Thus, the organoid size may be inversely correlated to sensitivity to both platinum-based chemotherapeutics and BPD-PDT, emphasizing that a characterization of tumor nodule growth may potentially explain differences in treatment susceptibility between organoid cultures of different cytologic origin.

### Pancreatic cancer organoids comprising cells of metastatic origin are resistant to OxPt chemotherapy

**Figure 2:**
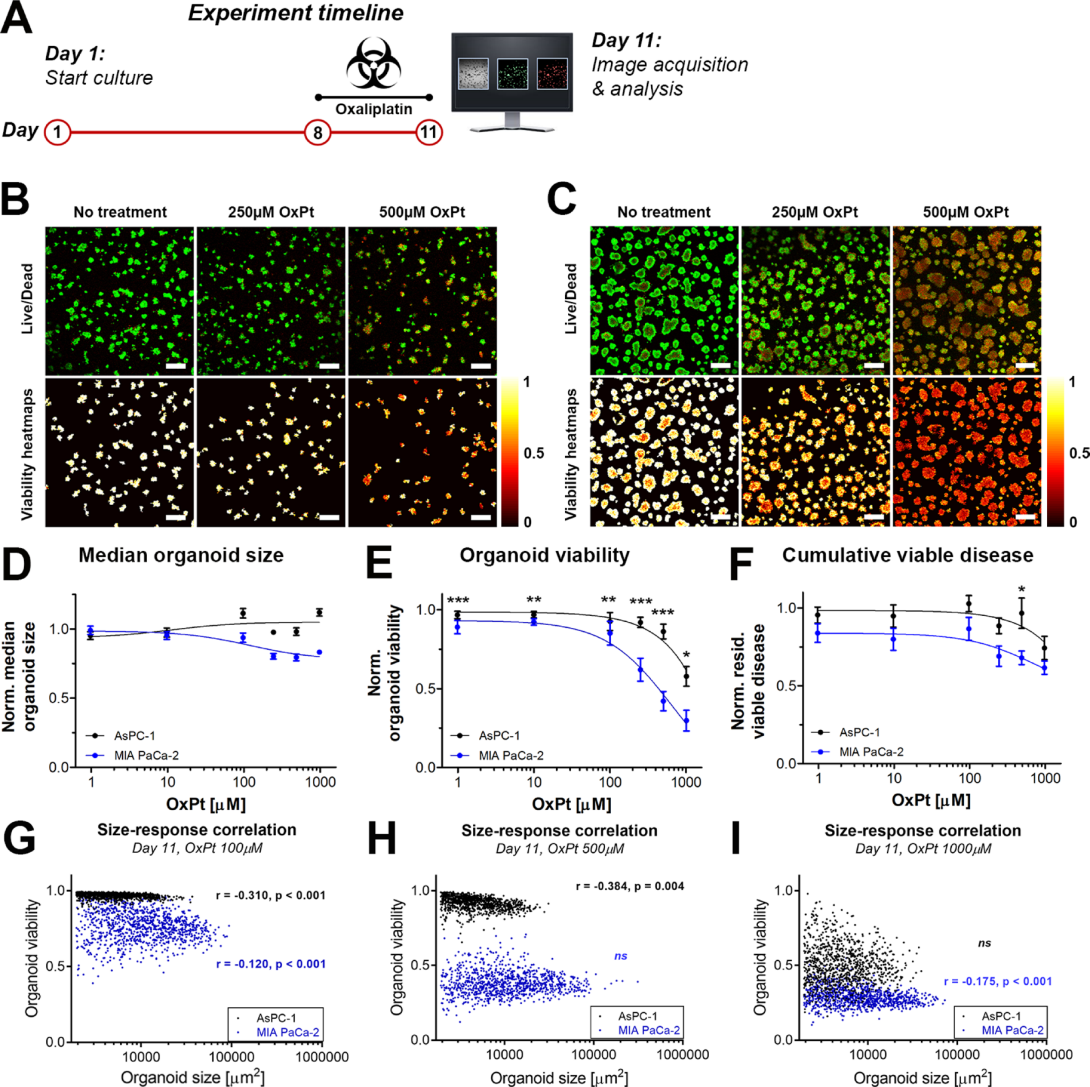
Comparison of OxPt treatment effects between AsPC-1 an d MIA PaCa-2 organoid cultures (**A**) Schematic representation of the experiment timeline. Live/dead fluorescence overlays and viability heatmaps of AsPC-1 organoids (**B**) and MIA PaCa-2 organoids (**C**) treated with OxPt. Scalebar = 400 μm. (**D**) Dose response curves based on the normalized median organoid size. (Mean ± SEM, *N* = 36). (**E**) Dose response curves based on the median viability of the organoid cultures. (Mean ± stdev *N* = 36). (**F**) Dose response curves based on the normalized cumulative viable area (Mean ± SEM, *N* = 9). (**G**–**I**) Scatter plots of organoid area and organoid viability following OxPt therapy on AsPC-1 (black) and MIA PaCa-2 (green) organoids. Size-response plots are depicted for the (G) 100 μM OxPT treatment, (H) 500 μM OxPt treatment, and (I) 1000 μM OxPt treatment. Significant size-response correlations are depicted in the image as determined using a Spearman’s ranked correlation test, displaying the Spearman coefficient r and the *p*-value for each data set (ns = not significant). For each data set, the first 1000 nodules are displayed within a single representative experiment.

As FOLFIRINOX constitutes one of the major types of palliative therapy for advanced metastatic cancer, we compared the susceptibility of the micrometastatic pancreatic cancer model (AsPC-1 organoids) and the primary tumor model (MIA PaCa-2 organoids) to OxPt, a core component of the FOLFIRINOX regimen. The experimental timeline for the OxPt dose-escalation experiment is depicted in Figure 2A. The live/dead fluorescence microscopy images and the corresponding viability heatmaps are shown for AsPC-1 and MIA PaCa-2 organoids in Figure 2B and 2C, respectively.

With respect to organoid area, OxPt exerted a slight dose-dependent decrease in size of the MIA PaCa-2 organoids that was not observed in the AsPC-1 cultures (Figure 2D). With respect to the viability, MIA PaCa-2 organoids were significantly more susceptible to OxPt compared to AsPC-1 (Figure 2E). Whereas the OxPt IC50 was fitted at 646 μM for MIA PaCa-2 organoid cultures under the given exposure conditions, the IC50 for AsPC-1 organoid cultures was >1 mM. Analysis of the cumulative residual viable disease per well following OxPt chemotherapy demonstrates that despite its ability to decrease viability, OxPt chemotherapy is inefficient in reducing the residual viable disease left within the cultures (Figure 2F). This parameter also demonstrates that AsPC-1 organoids are less affected by OxPt chemotherapy than the MIA PaCa-2 organoids, albeit to a minor extent and reaching significance only at a dose of 500 μM. Taken together, the results clearly demonstrate that the micrometastatic pancreatic cancer organoids are more resistant to OxPt chemotherapy in comparison to primary tumor organoids. The distribution of viability throughout the organoids suggest a homogeneous diffusion of OxPt throughout the organoids, thus corroborating previous findings [25, 26]. We identified negative correlations between organoid size and oxaliplatin sensitivity in both culture types for various concentrations, demonstrating that larger organoids were more susceptible to OxPt chemotherapy (Figure 2E–2I). This contradicts previous investigations that demonstrated a positive correlation between organoid size and cisplatin susceptibility in ovarian cancer models [27]. Our findings demonstrate reduced OxPt efficacy in AsPC-1 organoids compared to MIA PaCa-2 organoids even in nodules of comparable sizes (Figure 2E–2I), indicating that organoid size alone does not account for the OxPt resistance in AsPc-1 nodules. The potential origins of resistance were not further explored in this study, but may potentially be caused by alterations in adhesive and metabolic phenotypes [29, 30], two factors that have been implicated in treatment recalcitrance [30, 31].

### Neoadjuvant low-dose PDT acutely reduces residual viable disease following OxPt chemotherapy

**Figure 3:**
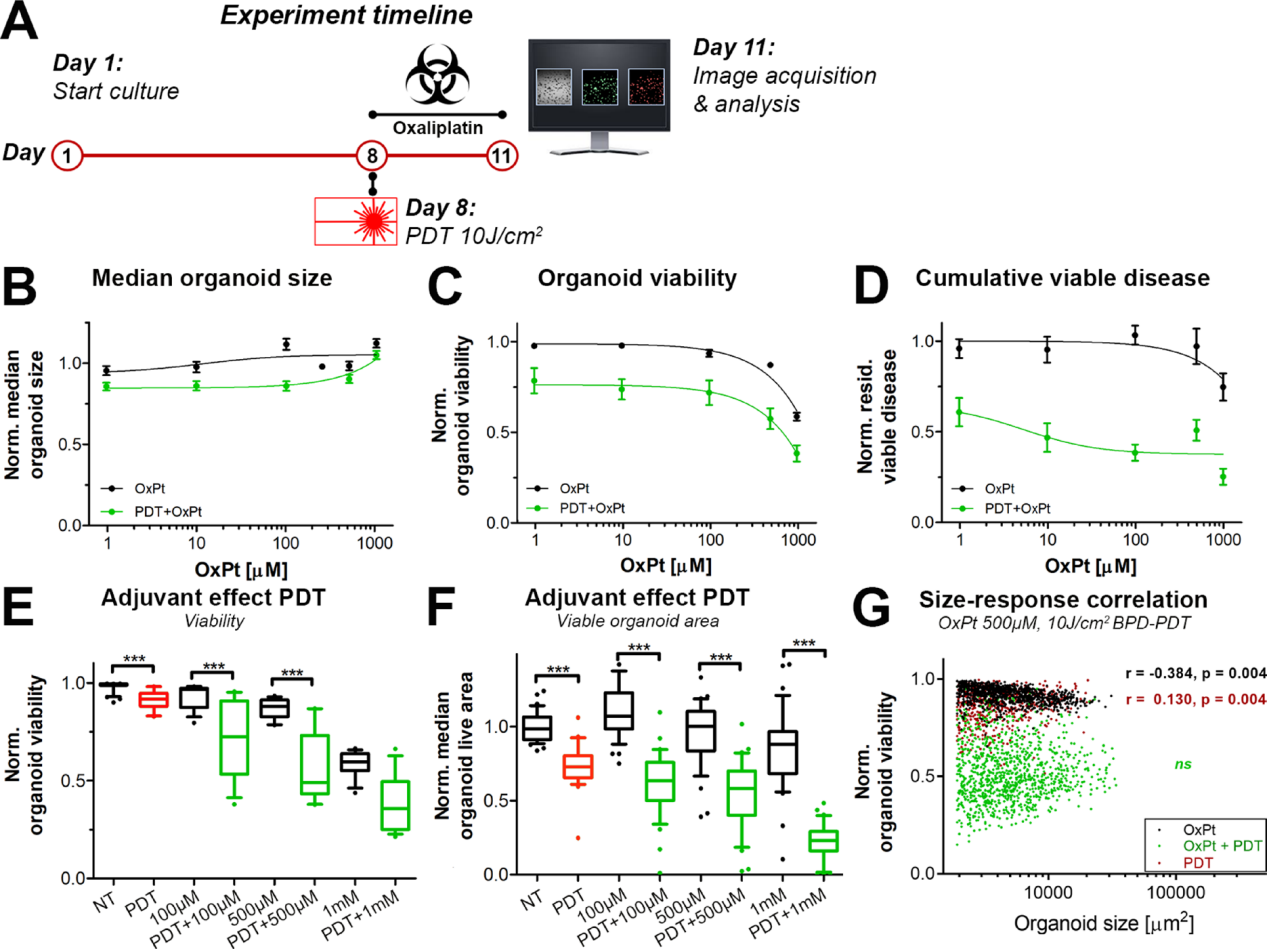
Comparison of immediate dose response effects of OxPt as a single therapy (black, same as Figure 2) or with neoadjuvant PDT (green) on AsPC-1 organoid cultures (**A**) Schematic overview of the experiment timeline. (**B**) Dose response curves based on the normalized organoid area (Mean ± stdev, *N* = 36). (**C**) Dose response curves based on the viability of the organoid cultures (Mean ± stdev, *N* = 36). (**D**) Dose response curves based on the cumulative viable area (Mean ± SEM, *N* = 9). Box-whisker plots depicting the medians, the 25th and 75th percentiles, and the 95% confidence interval of (**E**) the organoid viability (*N* = 36) and (**F**) the organoid live area per image (*N* = 36). (**G**) Scatter plots of organoid area and organoid viability following 500 μM OxPt (black), 10 J/cm^2^ BPD-PDT (red), and OxPt+PDT (green). Significant size-response correlations are depicted in the image as determined using a Spearman’s ranked correlation test, displaying the Spearman coefficient r and the *p*-value for each data set (ns = not significant). For each data set, the first 1000 nodules are displayed within a single representative experiment.

Various investigations have shown that PDT can overcome chemotherapy resistance in preclinical models [8, 9, 32–34]. To ascertain whether the efficacy of OxPt could be increased with neoadjuvant PDT, the OxPt dose-escalation experiment as depicted in Figure 2 was repeated, but with PDT performed immediately prior to treatment with OxPt (Figure 3A). A PDT regimen of 0.25 μM BPD (90 min photosensitization period) and 690 nm laser light irradiation at a radiant exposure of 10 J/cm^2^ (50 mW/cm^2^) was chosen as this roughly equated to an IC10 dose in terms of residual cumulative viable disease (Figure 3E).

There were no discernable effects of OxPt alone or the combination therapy on organoid size as determined using brightfield microscopy (Figure 3B). Viability was not affected by PDT alone (Figure 3E), but dropped significantly upon subsequent exposure to OxPt (Figure 3C), even at low OxPt doses. Approximations of IC50 values using the viability dose-response data were highly ambiguous. There was a dose-response correlation upon exposure to concentrations >100 μM, and viabilities were significantly lower in the PDT+OxPT treated groups compared to the groups receiving equimolar concentrations of OxPt alone, especially at lower OxPt doses (Figure 3E). The cumulative viable disease was significantly reduced by PDT alone, and the reduction was substantially enhanced by OxPt (Figure 3D and 3F). Based on the residual viable disease, the IC50 for OxPt alone could not be accurately estimated, but for PDT+OxPt the IC50 was approximated to 5.05 μM. It should be noted that the extent of viable disease was not reduced beyond 37% (bottom plateau). The adjuvant effect of PDT is best observed at the highest OxPt dose, where PDT+1000 μM OxPt reduced the median cumulative viable disease by 77.8% versus 30.0% by chemotherapy alone (*P <* 0.005) (Figure 3F). In contrast to the negative size-response correlation for OxPt, there was a positive correlation between organoid size and susceptibility to PDT that indicates that smaller organoids are significantly more susceptible to BPD-PDT compared to their larger counterparts. (Figure 3G). Importantly, these size-dependent responses to OxPt and PDT were lost in AsPC-1 cultures exposed to the combination therapy (Figure 3G). Thus, neoadjuvant PDT exerts a significant beneficial effect on the OxPt chemotherapy efficacy by mildly reducing overall organoid, reducing the extent of residual viable disease, and overcoming size-dependent susceptibilities.

We additionally explored whether the beneficial effects of neoadjuvant PDT on OxPt efficacy could be recapitulated on MIA PaCa-2 and PANC-1 organoid cultures. Early findings in 3D cultures of PANC-1 cells corroborate the findings observed in the AsPC-1 cultures, where low-dose PDT (2.5 J/cm^2^) significantly reduced residual viable disease following OxPt chemotherapy (1 μM, 72 h exposure) (Supplementary Figure 3). Moreover, there was a sequence-dependent interaction between OxPt and PDT; PDT performed before OxPt chemotherapy was significantly more effective than OxPt exposure before PDT (Supplementary Figure 3C), thereby corroborating previous findings on micrometastatic ovarian cancer organoids [21]. Results on MIA PaCa-2 organoids demonstrate that low-dose PDT (10 J/cm^2^) also exerted a significant beneficial effect on 100 μM OxPt exposure. These were evident upon assessment of residual viable disease, but not by evaluating organoid viability (Supplementary Figure 4). In conclusion, despite differences in treatment susceptibility in various cell lines, the beneficial effects of neoadjuvant PDT on OxPt chemotherapy efficacy can be recapitulated in various organoid cultures of pancreatic cancer cell lines, corroborating findings on combined PDT with carboplatin for treatment of ovarian cancer organoids [21].

### Prolonged efficacy of OxPt chemotherapy is augmented by neoadjuvant sublethal PDT

**Figure 4:**
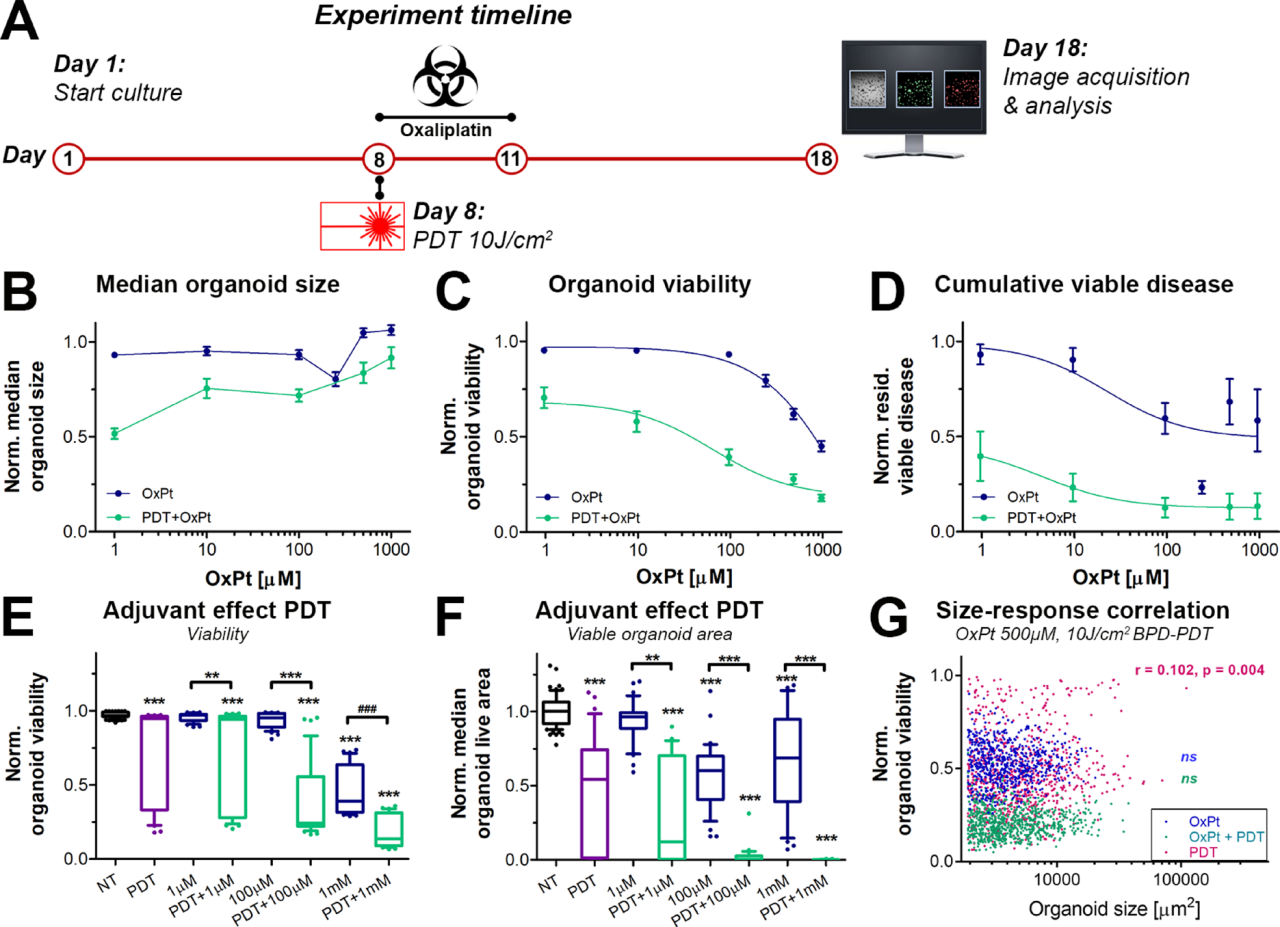
Effects of neoadjuvant PDT on prolonged OxPt dose response effects on AsPC-1 organoid cultures OxPt treatment groups are depicted in blue, PDT+OxPt treatment groups are depicted in turquoise, and PDT treatment alone is depicted in purple. (**A**) Schematic overview of the experiment timeline. (**B**) Dose response curves based on the normalized organoid area (Mean ± stdev, *N* = 36). (**C**) Dose response curves based on the viability of the organoid cultures (Mean ± stdev, *N* = 36). (**D**) Dose response curves based on the cumulative viable area (Mean ± SEM, *N* = 9). Box-whisker plots depicting the medians, the 25th and 75th percentiles, and the 95% confidence interval of (**E**) the organoid viability (*N* = 36) and (**F**) the organoid live area per image (*N* = 36). (**G**) Scatter plots of organoid area and organoid viability following 500 μM OxPt (blue), 10 J/cm^2^ BPD-PDT (purple), and OxPt+PDT (turqoise). Significant size-response correlations are depicted in the image as determined using a Spearman’s ranked correlation test, displaying the Spearman coefficient r and the *p*-value for each data set (ns = no tsignificant). For each data set, the first 1000 nodules are displayed within a single representative experiment.

Cancer relapse is frequently observed in pancreatic cancer patients following chemotherapy and is a major cause of mortality [35]. To investigate whether neoadjuvant PDT could prevent tumor regrowth, the OxPt dose response experiment was repeated with and without neoadjuvant PDT, after which the cultures were maintained in the absence of treatments for another week. On culture day 18, the experiments were terminated and the health of the cultures was assessed (Figure 4A).

Although median organoid sizes did not follow a dose-response correlation, organoids treated with PDT+OxPt appeared to be smaller in size compared to organoids treated with OxPt alone (Figure 4B), which is supported by similar findings in other tumor models [13, 36]. In terms of viability (Figure 4C), neoadjuvant PDT significantly enhanced the efficacy of OxPt chemotherapy. In combination with PDT, the OxPt IC50 was reduced from 1695 μM (95% CI: 735.7 to 3905 μM) to 66.95 μM (95% CI: 28.88 to 180.1 μM), representing a 25-fold enhancement in OxPt efficacy (*P <* 0.0001). PDT extensively reduced the extent of residual viable disease (Figure 4D), an effect that was further augmented by subsequent OxPt exposure. The fitted dose response curve for PDT+OxPt was significantly different from that of OxPt alone (*P <* 0.0001). Based on the residual viable disease, the IC50 of OxPt was 23.55 μM for the single therapy and 4.64 μM for the combination therapy PDT+OxPt. This relatively modest decrease in IC50 (5.1-fold efficacy enhancement) can be explained by the plateau in residual viable disease of OxPt alone; the bottom of the curve was at 0.49 of OxPt alone whereas it was at 0.12 for PDT+OxPt. Similarly to the results obtained on day 11 (Figure 3), larger organoids were less affected by PDT compared to their smaller counterparts, but there was no significant size-dependent response for the OxPt or the PDT+OxPt treatments (Figure 4G). Thus, PDT substantially enhances the OxPt efficacy by significantly reducing both organoid viability and cumulative viable disease on the long term, regardless of organoid size.

As a single therapy, PDT significantly reduced the viability of the AsPC-1 organoid cultures, giving rise to a heterogeneous mix of highly viable and non-viable organoids (Figure 4E, purple). Additional exposure to OxPt resulted in a more homogeneous distribution in viability, indicating that OxPt enhances PDT efficacy by reducing heterogeneity between organoids within the same culture. This heterogeneity is recapitulated when looking at the viable organoid area (Figure 4F). PDT as a single therapy yields a highly-dispersed range in the viable area of the organoids, which is strongly reduced upon subsequent OxPt exposure, even at low doses (e.g., 1–100 μM). Importantly, the PDT-OxPt combination therapy achieved near-complete eradication of the cultures at relatively low OxPt doses (100 μM), whereas 10-fold higher doses of OxPt alone left a substantial amount of residual viable disease. Together, the results unequivocally show that a combination of PDT and OxPt yields a superior therapeutic efficacy compared to each treatment alone, and both therapies harmonize to reduce inter-organoid heterogeneity and residual viable disease. Furthermore, the assessment of the prolonged effects of PDT and OxPt reveal a beneficial effect for the combination therapy to an extent that was not observed when treatment efficacies were evaluated at earlier timepoints, e.g., directly following OxPt exposure (day 10, Figure 3). These findings underscore the value of investigating treatment kinetics trough efficacy evaluations various time-intervals to fully grasp the scope of potential treatment effects.

### PDT exerts delayed treatment effects in a 3D culture model of micrometastatic pancreatic cancer

**Figure 5:**
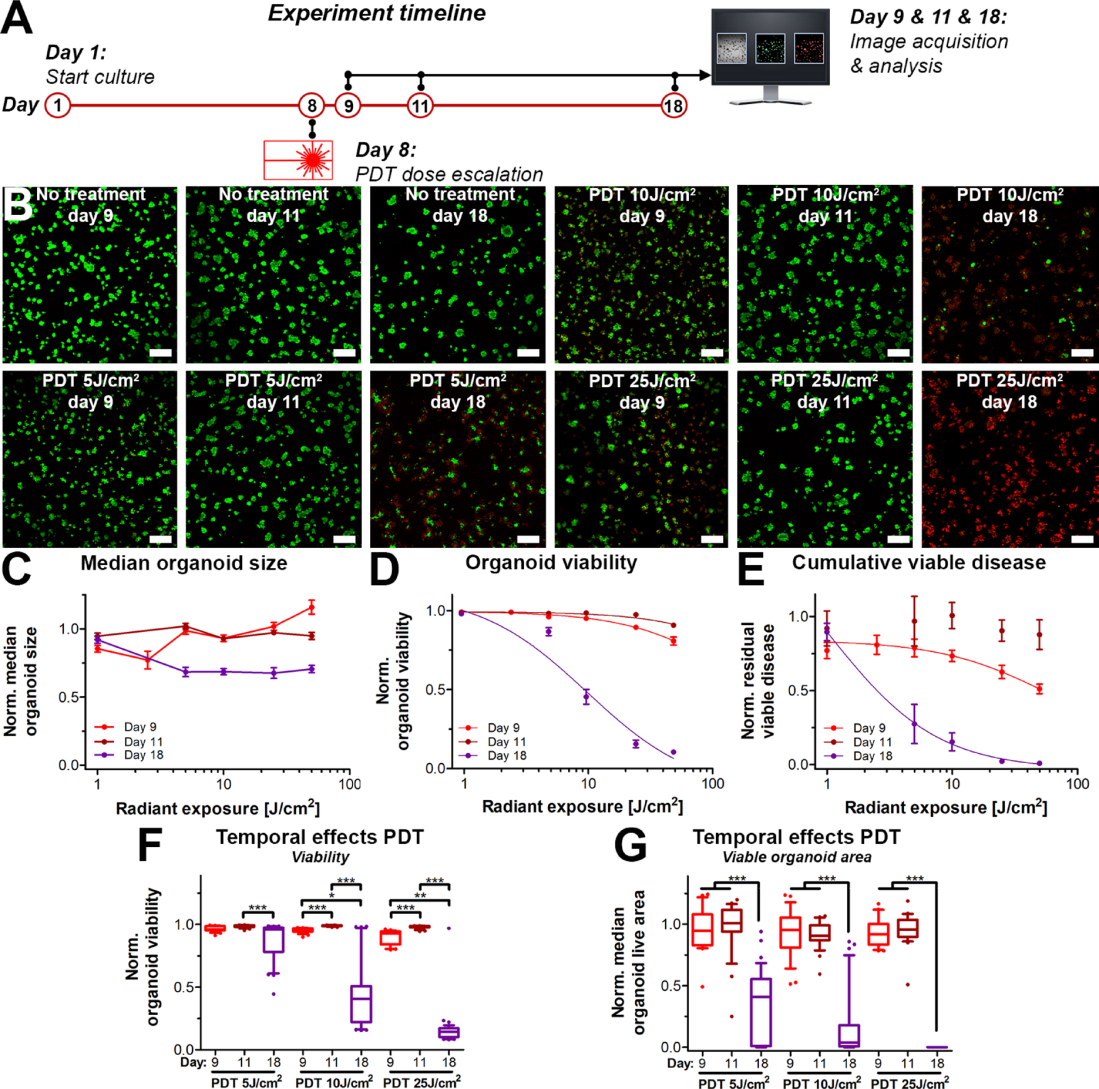
Temporal- and radiant exposure-dependent effects of PDT on AsPC-1 organoid cultures as determined on day 9 (red), day 11 (dark red) and day 18 (purple) (**A**) Schematic overview of the experiment timeline. (**B**) Live/dead fluorescence overlays of the cultures following PDT at the indicated radiant exposures. Live cells are depicted in green, dead cells are shown in red. Scalebar = 400 μm. (**C**) Dose response curves based on the normalized organoid area (Mean ± stdev, *N* = 24–36). (**D**) Dose response curves based on the viability of the organoid cultures (Mean ± stdev, *N* = 24–36). (**E**) Dose response curves based on the cumulative viable area (Mean ± SEM, *N* = 6–9). Box-whisker plots depicting the medians, the 25th and 75th percentiles, and the 95% confidence interval of (**F**) the organoid viability per image (*N* = 24–36) and (**G**) the organoid live area per image (*N* = 24–36).

The observation that PDT strongly reduced the extent of the viable disease following prolonged culturing post-treatment (day 18) when compared to the immediate response (day 8 and day 11) prompted the investigation towards the temporal effects of PDT. The effects of PDT have classically been viewed as relatively immediate (24–48 h), where the short-lived reactive molecular species cause immediate oxidation of biomolecules, followed by direct necrosis or delayed cell death through apoptosis [36–41]. The observations in Figures 3 and 4 indicate that in 3D cultures, the effects of PDT on these 3D cultures may be much more prolonged than generally assumed. We therefore investigated the temporal effects of PDT alone by examining the organoid cultures at varying time intervals following treatment (Figure 5A).

Temporally spaced live/dead imaging following the PDT dose-escalation experiments reveal dramatic changes in the extent of viable disease of cultures analyzed on day 9 and 11 versus day 18 (Figure 5B). Again, organoid size (Figure 5C) did not differ between the PDT radiant exposure doses and analysis time-points. (Figure 5D) Analysis of viability revealed only slight dose-response correlations on day 9 (IC50 216 J/cm^2^) and day 11 (ambiguous IC50), yet the effects of PDT demonstrated effective dose-dependent organoid killing on day 18 (IC50 12 J/cm^2^). (Figure 5E) The residual viable disease followed a dose-response correlation immediately following PDT (Day 9), achieving a 10% reduction in residual viable disease at a dose of 10 J/cm^2^, justifying the use of this dose for the combination therapy experiments. However, the dose-response correlation was lost when cultures were analyzed on day 11, as the cultures appeared to have recovered from the initial treatment on day 8. Contrastingly, yet similar to the viability assessment, there was a strong dose-dependent reduction in viable disease on day 18. The IC50 for radiant exposure was significantly lower on day 18 (1 J/cm^2^) compared to day 9 (58 J/cm^2^), whereas no curve could be fitted for the day 11 analysis. PDT dose responses of the cultures on day 9 and 11 were not significantly different. In contrast, the responses measured on day 18 differed with high statistical significance from the responses measured on day 9 and 11 (Figure 5F, 5G), thus revealing delayed PDT responses in these 3D cultures.

## DISCUSSION

It is generally accepted that combination treatments are needed for effective cancer therapy, FOLFIRINOX being an important example with respect to pancreatic cancer. These combinations are likely to be most effective when the therapeutic modalities have different modes of action and non-overlapping toxicity to healthy tissue. BPD-PDT constitutes a biophysical therapy that causes massive oxidative cellular damage and bypasses the barriers to apoptosis by direct destruction of mitochondrial membranes and release of cytochrome C [42]. As OxPt, part of FOLFIRINOX, achieves tumor destruction via DNA crosslinking [43], the combination of the mechanistically distinct PDT and OxPt constitutes a translationally relevant combination therapy for advanced pancreatic cancer. Therefore, this study investigated the adjuvant effects of PDT on the efficacy of OxPt chemotherapy. Our findings demonstrate decreased OxPt efficacy in a micrometastatic pancreatic cancer model compared to an equivalent model comprising cells from a primary tumor line. Neoadjuvant low-dose PDT significantly increased the efficacy of OxPt, which was further augmented in a time-dependent manner. These findings were mostly attributed to the delayed treatment effects of PDT on this model, relative to the time and dose-dependent effects of PDT and combinations observed in other models [13, 21, 39, 44].

The results of this study correlated well with previous findings on the BPD-PDT-susceptibility of PANC-1 organoids [27], and MIA PaCa-2 organoids [19]. Although the AsPC-1 organoids in the current study demonstrated substantial PDT-resistance in comparison to previous findings, these can be attributed to subtle differences in the experimental design [27] as it was previously demonstrated that variations in PDT dose parameters can have a great impact on the treatment outcomes [22, 39]. However, our findings on the effects of PDT on organoid cultures of PanCa contrasts with previous findings reported for ovarian cancer organoids. Whereas carboplatin diffusion and therapeutic efficacies were confined to the periphery of OVCAR-5 organoids [21], the OxPt effects were homogeneous throughout the AsPC-1 and MIA PaCa-2 nodules. Moreover, PDT was shown to disrupt ovarian cancer organoids leading to the disassociation of cancer cells from the nodules [21], whereas the effects of PDT on 3D culture of PanCa cells were confined to the periphery of the organoids. These discrepancies can be attributed to the different origins of the cell types and the distinct architectures of the organoids they form in 3D cultures. Such results highlight that cancer therapies have highly divergent mechanisms of action on different organotypic models of cancer.

The ability of PDT to enhance chemotherapy efficacies has been well-documented [21, 33, 44–46], and was shown to range from synergism to antagonism depending on the therapy sequence, cell lines, and photochemotherapy combination [20]. By employing a 3D model for micrometastatic ovarian cancer, we previously showed that pretreatment of the 3D cultures with BPD-PDT synergistically enhanced carboplatin efficacy, whereas the reverse sequence (carboplatin first, then BPD-PDT) did not [21]. Similar treatment effects were also observed in the current study (Supplementary Figure 3). Thus, the development of combinatorial regimens to improve overall response is not simply a matter of combining two mechanistically distinct therapies, and emphasizes the need for physiologically-relevant models to assess combinations.

The beneficial effects of PDT on the OxPt efficacy likely stems from the distinct cytotoxic mechanisms of both therapies. PDT causes oxidation of vital biomolecules that culminates in the direct induction of apoptosis [47], and severely disrupts cellular redox homeostasis in surviving cells [7]. Subsequent OxPt-induced DNA damage may be more efficient as metabolism may be impaired and cells may perish through severe genomic instability combined with imbalanced redox homeostasis. Additionally, the multidrug transporter ABCG2 has been shown to be downregulated following PDT in orthotopic *in vivo* models of pancreatic cancer, leading to increased irinotecan retention and therapeutic efficacy [9]. A recent investigation suggested that both mechanisms may be responsible for increased OxPt sensitivity of cancer cells following PDT, as was demonstrated on colorectal cancer cell lines (2D cultures) following hypericin PDT [48]. In addition, PDT is agnostic to the chemoresistant status of cells as gemcitabine-resistant pancreatic cancer cells retained sensitivity to PDT in 2D cultures [8]. Moreover, organotypic cultures of stable oxaliplatin-resistant pancreatic cancer cells exhibited altered 3D architecture and increased sensitivity to BPD-PDT relative to organoid cultures of the drug-naive cells [17].

The current study demonstrates the capacity of 3D cultures to comprehensively assess mesoscopic treatment effects that cannot be captured in 2D cell cultures or in *in vivo* models. As 3D cultures can be maintained for prolonged periods of time compared to 2D cultures, the organoid cultures enabled us to take temporal treatment effects into consideration. Consequently, we identified unique treatment effects of PDT, OxPt, and the combined therapy on tumor architecture, and revealed a prolonged interaction between neoadjuvant PDT and OxPt on these cultures. The combined treatment was shown to overcome the limitations of both PDT (high residual heterogeneity and viability) and OxPt (high residual viable disease); the combination therapy PDT+OxPt yielded a homogeneously dispersed population of organoids with low viabilities and viable volumes. These findings highlight the importance of kinetic studies when evaluating combination therapies. Appropriate timing of treatment analysis is thus crucial to discern the full scope of possible treatment effects.

Although it is subject for further investigations, the results of this study provide a promising indication for PDT in the clinical management of advanced pancreatic cancer. The use of an ascites-derived cell line in an adherent 3D culture model bears resemblance to the adhesion of multicellular spheroids to the peritoneal wall or the mesothelial layers of various organs. This has particular significance for pancreatic cancer, as peritoneal invasion is frequently observed following surgery with curative intent. Survival rates for patients with peritoneal carcinomatosis are dismal, and procedures to prevent it are scarce [49]. Given the outcomes of the current investigation, a combined therapy of PDT and OxPt may hold potential to combat peritoneal pancreatic cancer metastases following surgery. A recent investigation demonstrated the utility of PDT for the treatment of micrometastatic ovarian cancer *in vivo*, using a photoimmunoconjugate composed of BPD and cetuximab and light-diffusing fiber tips for the complete irradiation of the abdominal cavity, achieving significantly reduced micrometastatic tumor burdens [49]. Intraperitoneal chemotherapy yields promising clinical outcomes for peritoneal carcinomatosis of various cancers [50–53]. The current investigation represents a preclinical model of peritoneal metastases of pancreatic cancer in which the combination of PDT with OxPt shows promise as a clinically feasible strategy to reduce metastatic burdens and prevent peritoneal carcinomatosis following surgery.

A combination therapy of BPD-PDT and the FOLFIRINOX regimen may prove to be an efficient strategy for pancreatic cancer treatment. Within the FOLFIRINOX regimen, halting of DNA replication by irinotecan (topoisomerase inhibition) was shown to work in synergy with the DNA damaging therapies of folinic acid and 5-fluoruracil (base-substitution) and OxPt (DNA crosslinking) [1]. A critical aspect explaining the success of FOLFIRINOX is the relative absence of overlapping toxicities of its individual components, caused by the different molecular targets of each chemotherapeutic agent. In this respect, BPD-PDT is a suitable candidate for combination with FOLFIRINOX as it mainly targets the mitochondrial membranes [35], and thus lacks any overlap in toxicity profile with the chemotherapeutic agents. This study and others have demonstrated that PDT amplifies, and benefits from, the treatment effects of the individual components of the FOLFIRINOX regimen [9, 48, 54]. Lastly, the results of this study demonstrated that a combination therapy of PDT and OxPt overcomes the organoid size-dependent responses of each individual therapy. These findings warrant further exploration towards the beneficial effects of BPD-PDT in a neoadjuvant setting to the clinically employed regimen of FOLFIRINOX in mouse models of advanced pancreatic cancer.

In conclusion, as single therapies typically fail to achieve satisfactory clinical outcomes for advanced pancreatic cancer, it is believed that a combination of therapies with distinct modes of action can provide better disease management without overlapping toxicities to healthy tissue. Both PDT and FOLFIRINOX have different mechanisms of action and show promising clinical results as individual therapies. The results from this study demonstrates that a combination of these therapies was significantly more effective compared to either therapy alone, using organoid models of pancreatic cancer cell lines of either metastatic or primary tumor origin. These findings demonstrate the promise of this combination therapy in the management of advanced pancreatic cancer. Neoadjuvant PDT may constitute a treatment option for surgery-ineligible patients regardless of performance score, given that lower chemotherapy doses could achieve effective disease management. As technical advancements pertaining to light administration throughout the abdominal cavity are underway, PDT may be employed as a post-operative procedure to prevent peritoneal carcinomatosis following surgery, of which the current study provides promising preclinical evidence. The use of 3D models with computed analysis of treatment outcomes allows testing of large numbers of combinations, which are necessary for establishing the most effective set of treatment conditions.

## MATERIALS AND METHODS

### Cell culture

Human pancreatic cancer cell lines MIA PaCa-2, PANC-1, and AsPC-1 were obtained from the American Type Culture Collection (ATCC, Manassas VA, obtained between 2010 and 2014). MIA PaCa-2 and PANC-1 cells were maintained in Dulbecco’s modified Eagle’s medium (DMEM, Corning, Tewksbury MA) supplemented with 10% (v/v) fetal bovine serum (Gibco, ThermoFisher, Waltham MA), 1% (v/v) penicillin/streptomycin (Corning). Cells were typically passaged weekly at 1:10 ratio. AsPC-1 cells were cultured on Roswell Park Memorial Institute 1640 (RPMI, Corning), supplemented with 10% (v/v) fetal bovine serum and 1% (v/v) penicillin/streptomycin. AsPC-1 cultures were passaged weekly at a 1:8 ratio. Cells were used for experimentation between passages 5 and 30. Cells tested negative for mycoplasma following completion of the study (Mycoalert plus, Lonza, Morristown NJ).

To establish 3D adherent organoid cultures, AsPC-1 or MIA PaCa-2 cells were seeded on solidified growth factor-reduced Matrigel (Corning) in black-walled 24-wells plates (Sensoplate, Greiner Bio-One, Monroe, NC), at a density of 7500 cells/well (1 mL per well). Cultures were maintained in complete culture medium, additionally supplemented with 2% (v/v) growth-factor reduced Matrigel. Matrigel lot numbers used throughout this study were 5173009 and 36819 and contained 9.2 mg/mL protein and <1.5 U/mL endotoxin. Culture media, supplemented with 2% Matrigel was refreshed every 3–4 days.

### Photodynamic therapy

The 3D cultures were subjected to PDT on culture day 8. Cultures were incubated with 0.25 μM BPD (Sigma Aldrich, St. Louis MO) in fresh culture medium for 90 min. Subsequently, the BPD-supplemented medium was replaced with fresh culture 2% Matrigel-containing medium and cultures were irradiated with 690 nm laser light (Intense, North Brunswick NJ), at a power density of 50 mW/cm^2^, and at total radiant exposures ranging between 1–50 J/cm^2^. Treatment effects were assessed on culture day 9, 11 and 18 as described below.

### OxPt chemotherapy

The 3D cultures were treated with OxPt (Selleck Chemicals, Houston TX) on culture day 8 for a duration of 72 h (to culture day 11). After OxPt incubation, cultures received fresh 2% Matrigel-containing medium devoid of OxPt until the termination of the experiments. Treatment effects were assessed on culture day 11 or 18 as described below.

### Quantitative assessment of culture growth and treatment effects

Organoids were stained *in situ* using 2 μM calcein AM (ThermoFisher) and 3 μM propidium iodide (PI, Sigma-Aldrich) for 30 min, after which the cultures were imaged on an Olympus FV1000 confocal laser scanning microscope at a 4× magnification, 4 μs pixel dwell time, and a resolution of 512 × 512 px. Calcein and PI fluorescence was detected at λ_exc_= 488 nm and λ_em_= 520 ± 20 nm, and λ_exc_= 559 nm and λ_em_= 630 ± 20 nm, respectively. Images were subsequently analyzed using the CALYPSO methodology as described previously [19], applying a size threshold of 50 px (1925 μm^2^ or circa 6 clustered cells). In every experiment, four consecutive non-overlapping images were taken per well, containing 50–80 individual tumor organoids per image. Sizes, viabilities and viable organoid areas were determined for every individual tumor organoid, and the depicted data represents the median or average value per image as indicated. Cumulative viable disease was determined by summing the viable organoid areas per well (4 images/well, 3 wells/experiment), and normalizing the values to the no treatment controls.

Quantitative analysis of culture growth was performed using the CALYPSO methodology [19] without live/dead staining. Adaptive thresholding on the brightfield images was utilized to extract the sizes of individual tumor organoids at different time points.

### Statistical analysis

All statistical analyses were performed in Graphpad Prism 5.0 (La Jolla, CA). Data were tested for normality using the Pearson-Omnibus test. Non-Gaussian data were analyzed using either a Mann-Whitney *U*-test, or a Kruskal-Wallis and Dunn’s post-hoc test for multiple comparisons. Gaussian data were analyzed using either student’s *t*-test or a one-way ANOVA and Bonferroni post-hoc test for multiple comparisons. Significance is indicated as either single asterisk (*P <* 0.05), double asterisk (*P <* 0.01), or triple asterisk (*P <* 0.005). Unless otherwise indicated, asterisks refer to significant difference between the control group and the designated group. Size-response correlations were calculated using a Spearman’s rank test.

## SUPPLEMENTARY MATERIALS FIGURES



## Abbreviations

OxPt: oxaliplatin
BPD: benzoporphyrin derivative
PDT: photodynamic therapy
PI: propidium iodide
