# Supplementary material for: Neoadjuvant photodynamic therapy augments immediate and prolonged oxaliplatin efficacy in metastatic pancreatic cancer organoids
Source: Oncotarget. 2018 Feb 6;9(16):13009–22. doi: 10.18632/oncotarget.24425 (PMC5849191; doi:10.18632/oncotarget.24425)
Supplement: Supplementary file 1 [file oncotarget-09-13009-s001.pdf]

Neoadjuvant photodynamic therapy augments immediate and prolonged oxaliplatin efficacy in metastatic pancreatic cancer organoids

SUPPLEMENTARY MATERIALS

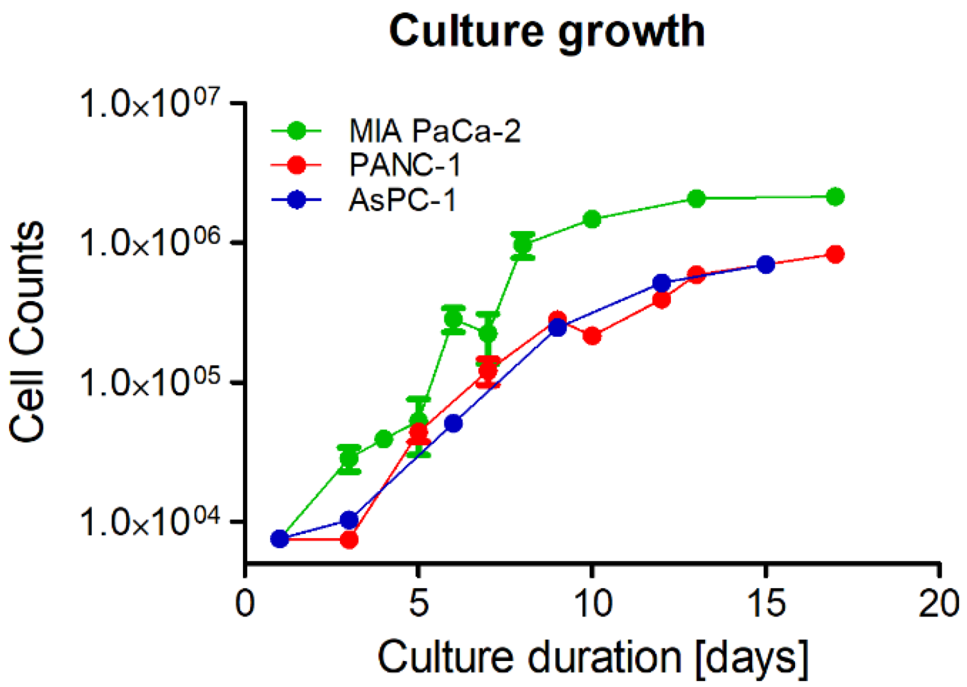

Supplementary Figure 1: Assessment of the growth of pancreatic cancer cell lines in adherent 3D cultures. Cultures were harvested at the indicated culture duration by removing cell culture media and adding 600 µL Dispase (Sigma-Aldrich). After 2 h of incubation at 37° C, 1 mL culture medium was added and cell suspensions were collected and centrifuged (5 min, 1100 g). Cell pellets were resuspended in phosphate buffered saline and counted in duplicate using a hemocytometer. Depicted are the mean ± SEM of $N = 2–6$ counts. Cell counts for MIA PaCa-2, PANC-1, and AsPC-1 lines are given in green, red, and blue, respectively.

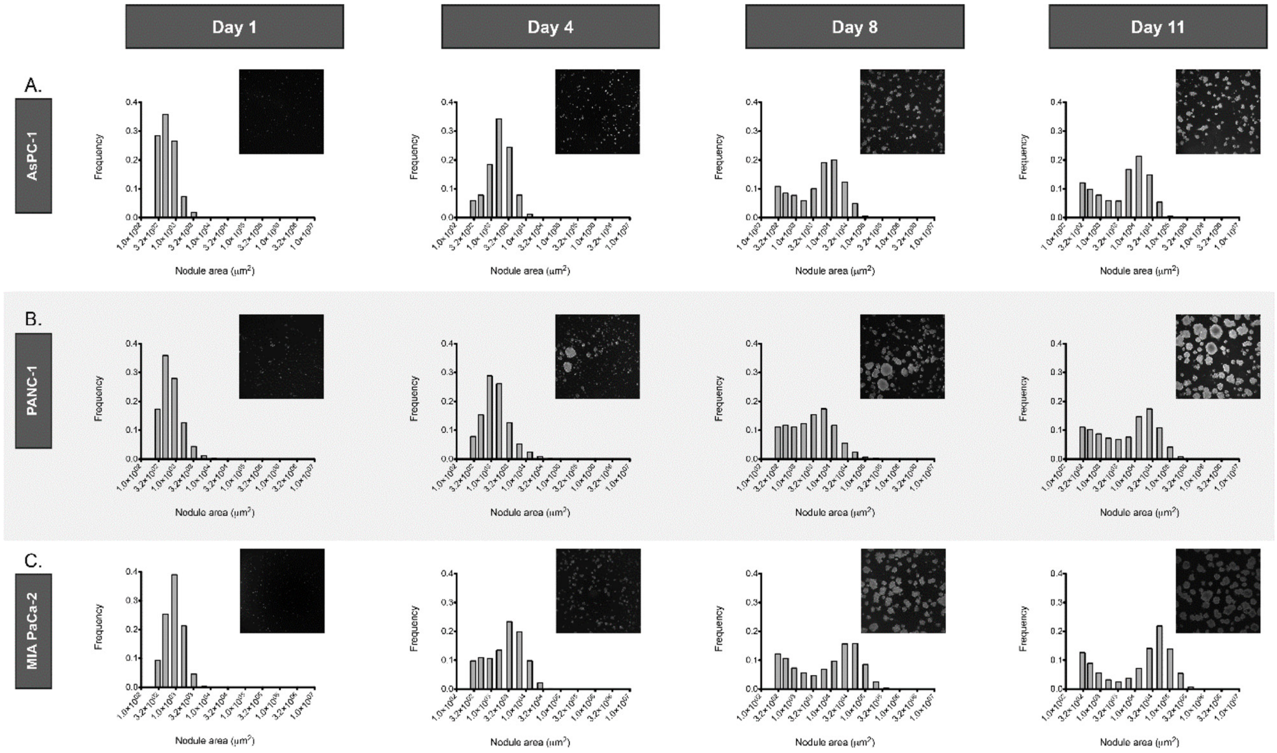

Supplementary Figure 2: Assessment of organoid growth and size distribution for pancreatic cancer cell lines grown as adherent 3D cultures. Cultures were imaged using dark-field microscopy (Zeiss Axiovert), and organoid sizes were extracted using the image analysis methodology described previously (Celli *et al.*, 2010). Depicted are the lognormal size distributions for AsPC-1 (**A**), PANC-1 (**B**), and MIA PaCa-2 cells (**C**).

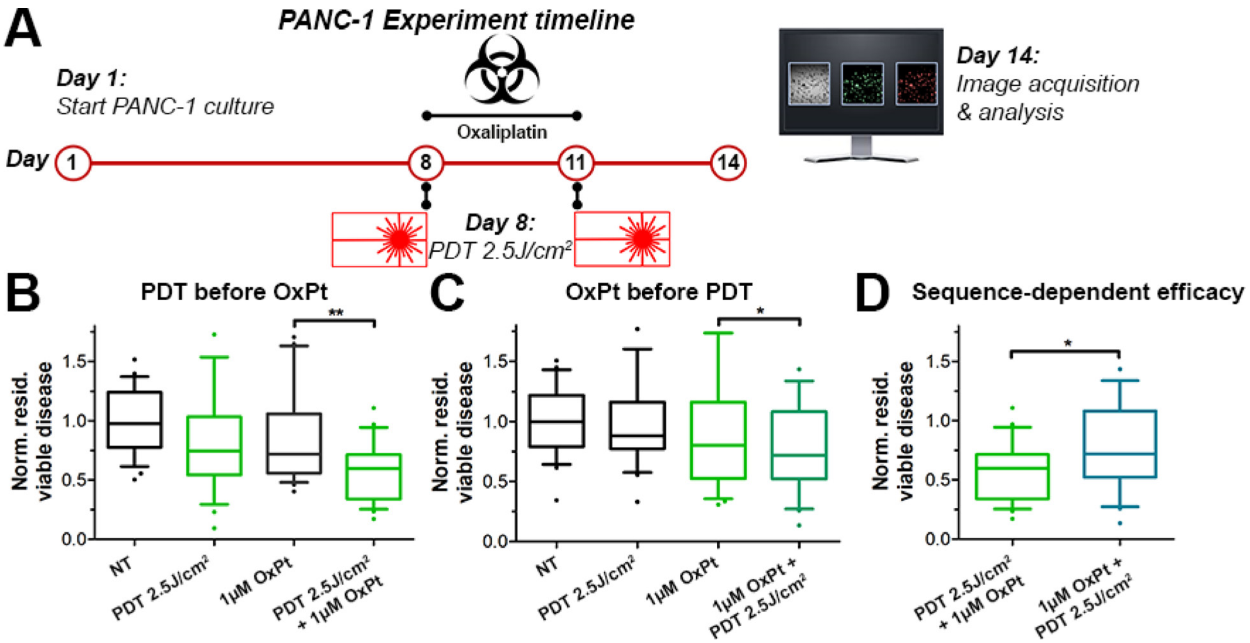

Supplementary Figure 3: Effects of PDT+OxPt photochemotherapy on PANC-1 organoid cultures. (**A**) Experiment timeline, depicting initiation of the PANC-1 cultures on day 1, OxPt exposure from day 8 to 11, and PDT performed either before (day 8) or after OxPt treatment (day 11). Treatment effects were assessed on day 14, where images were acquired on a Zeiss Axioobserver Z1 and image analysis was performed in accordance with the qVista methodology (Celli *et al.*, 2014). (**B**) Normalized cumulative viable disease in PANC-1 organoid cultures following no treatment, PDT (0.25 µM BPD, 90 min photosensitization, 2.5 J/cm^2 at 50 mW/cm^2) on culture day 8, OxPt chemotherapy (1 µM OxPt for 72 h), or combination therapy (PDT on day 8, immediately followed by OxPt chemotherapy). Data passed the D'Agostino & Pearson-Omnibus normality test and was analyzed using a student's *t*-test. (**C**) Box-whisker plots depicting the medians, the 25th and 75th percentiles, and the 95% confidence interval of PANC-1 organoids receiving no treatment, OxPt chemotherapy (1 µM OxPt for 72 h from day 8 to day 11), PDT (0.25 µM BPD, 90 min photosensitization, 2.5 J/cm^2 at 50 mW/cm^2) on culture day 8, or underwent combination therapy (OxPt chemotherapy from day 8 to day 11, immediately followed by PDT). Data did not pass the D'Agostino & Pearson-Omnibus normality test and was analyzed using a Mann-Whitney-*U*-test. (**D**) The residual viable disease after PDT and subsequent OxPt chemotherapy (green) was directly compared to a combination regimen consisting of OxPt chemotherapy and subsequent PDT (turquoise). Data passed the D'Agostino & Pearson-Omnibus normality test and was analyzed using a student's *t*-test.

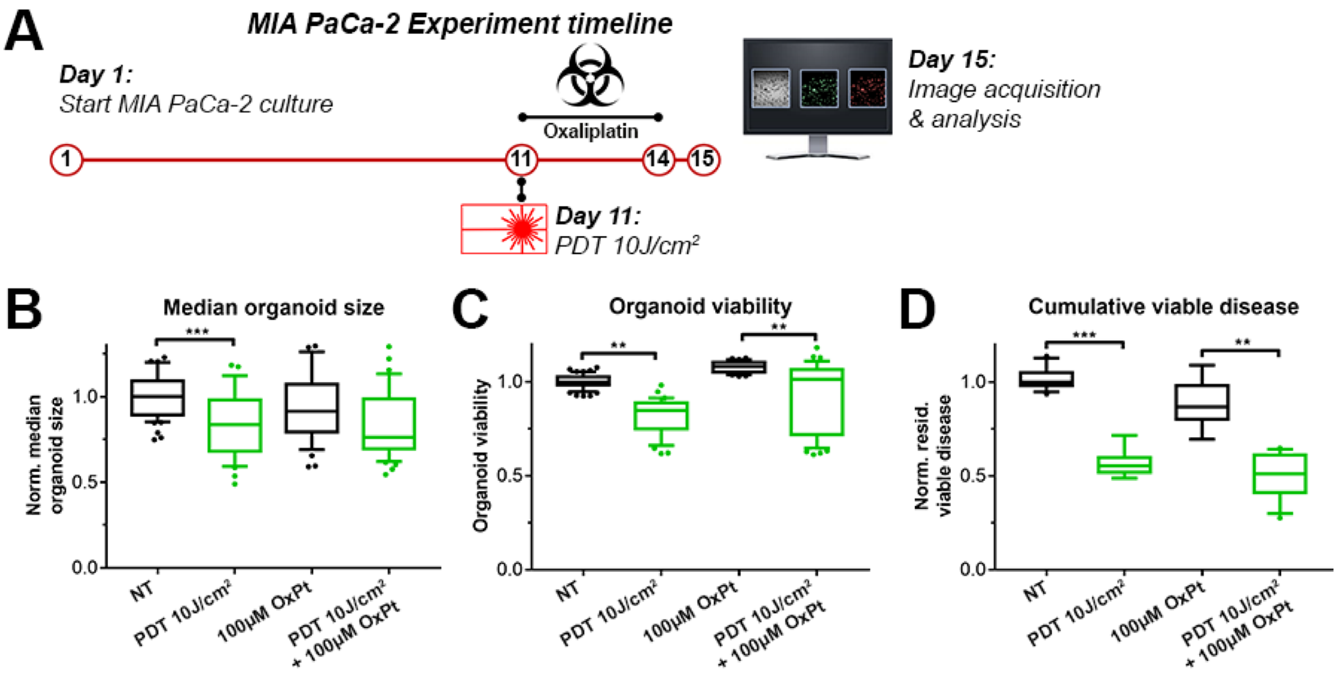

Supplementary Figure 4: Effects of neoadjuvant PDT (10 J/cm², 150 mW/cm², 60 min photosensitization with 0.25 µM BPD) on the efficacy of 100 µM OxPt in MIA PaCa-2 organoid cultures. (**A**) Schematic overview of the experiment timeline, depicting initiation of the cultures on day 1, exposure to PDT on day 11, OxPt treatment from day 11 to day 14 (72 h), and assessment of treatment response on day 15. (**B**–**D**) Box whisker plots depicting the median, 25th and 75th percentile and 90% CI of the normalized MIA PaCa-2 organoid area following treatment ($N \geq 36$). (C) Box whisker plots depicting the median, 25th and 75th percentile and 90% CI of the normalized viability of the MIA PaCa-2 organoids following treatment ($N \geq 36$). (D) Box whisker plots depicting the median, 25th and 75th percentile and 90% CI of the normalized residual viable disease in the MIA PaCa-2 organoid cultures following treatment ($N \geq 9$). All statistical analyses were performed using a Kruskal-Wallis test and Dunn's post-hoc test.
